# Investigation of the synergistic effect of nonionic surfactants on emulsion resolution using response surface methodology

**DOI:** 10.1039/d2ra04816g

**Published:** 2022-10-28

**Authors:** Sofiah Atirah Raya, Ismail Mohd Saaid, Aminah Qayyimah Mohd Aji, Ahmad Amirhilmi A Razak

**Affiliations:** Department of Petroleum Engineering, Universiti Teknologi PETRONAS 32610 Bandar Seri Iskandar Perak Malaysia sofiahatirah@gmail.com ismailsaaid@utp.edu.my aminah_19001042@utp.edu.my; Petronas Research Sdn. Bhd., Jln Ayer Hitam, Kawasan Institusi Bangi 43000 Bandar Baru Bangi Selangor Malaysia

## Abstract

The production of crude oil is always accompanied by water production, which may create severe separation problems. It is important to understand the stabilization mechanism and parameters contributing to the formation of emulsion, specifically the synergy mixing of surfactants. These factors have not been studied primarily in previous studies. The main objective of the current work was to assess the influence of synergy mixing of nonionic surfactants, sorbitan monooleate (hexitol) and polysorbate 80 (glycol), which are mainly affecting the stability of oil-in-water emulsions. Several factors, such as the mixing rate, mixing time, and aging time of the studied emulsions were also investigated. Response surface methodology (RSM), and central composite design (CCD) were employed to design the experiments. Emulsion stability was measured through a static bottle test over a range of time (1–7 days) at a temperature of 60 °C. A model was established with a coefficient of determination value at 0.8814 and the highest emulsion stability achieved was 42.83%. The least water separation was observed at 0.5 v/v% hexitol, 1.5 v/v% glycol, 15 000 rpm mixing rate in 5 minutes, and seven-day ageing time to achieve ∼41.56% emulsion stability. The minimum emulsion stability of ∼25.0% was observed using 0.5 v/v% of sorbitan monooleate and polysorbate 80 at 5000 rpm of mixing rate in 15 min and under seven days of observation. The results also revealed that the mixing time and ageing time do not affect the stability of the prepared emulsions. Hexitol, mixing rate, synergy mixing of nonionic surfactants and polysorbate 80, and mixing speed significantly influence emulsion stability. The *R*^2^ value of 88.14% verified that the model is well-fitted and the optimal values for the input variables were successfully obtained using RSM.

## Abstract 2

Response surface methodology (RSM) plot of formulation optimization showing the effect of variables on hexitol and glycol. Response surface plots of the predicted stability as a function of hexitol and glycol.

## Graphical abstract

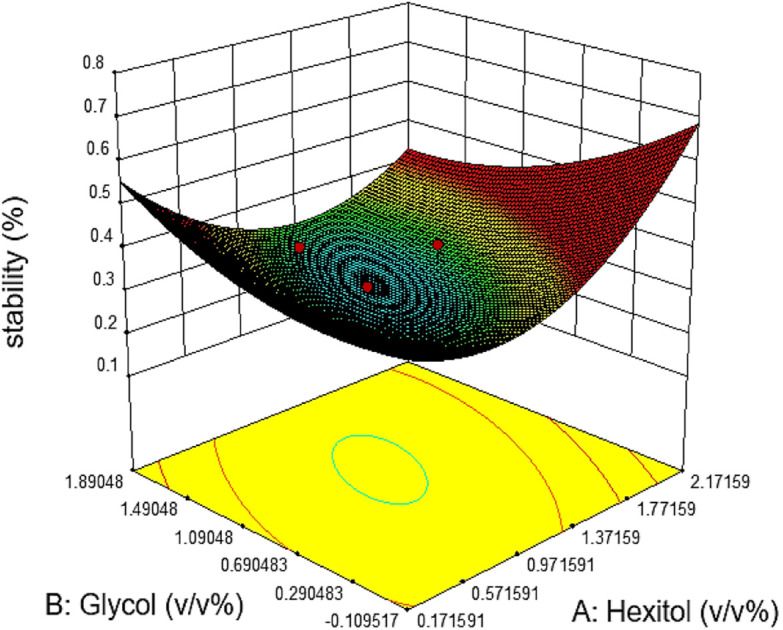

## 1 Introduction

Emulsions are metastable systems typically formed in the presence of surfactant molecules, amphiphilic polymers, or solid particles. Thermodynamically, an emulsion is an unstable system, and several circumstances, such as creaming, flocculation, Ostwald ripening, and coalescence will slowly affect the emulsion.^1^ Under conditions where a two-phase system comprises two liquids not forming a homogeneous solution when mixed, one is (dispersed phase) constantly dispersed as globules in the second phase (continuous phase); this is called dispersion.^2,3^ The large area of the interface between the mixture of the two immiscible liquids must be maintained; emulsions are thermodynamically unstable.^4^

During the extraction and transportation of crude oil, the formation of an emulsion is undeniable. The formation occurs once the heterogonous mixture flows in the piping valves and porous rocks and endure turbulence at high temperature or high pressure. The primary reasons for enhancing the emulsion formation include surface-active agents, ionic compositions, and the pH of the water. Emulsions generate significant operational and environmental challenges in industries, specifically in the petroleum industry. They trigger flow assurance difficulties due to high viscosity, high shear rates, and turbulence zones at different production facilities.^5^ The presence of water in crude oil leads to unwanted consequences, such as corrosion, rise in conductivity, and leaching of additives. The elimination of highly stable oil droplets in the produced water encountered difficulties in offshore production due to their long residence time and the requirement of large equipment volumes.^6^

The emulsions were also reviewed as special liquid-in-liquid colloidal dispersions. The kinetic stability of the emulsions depends on their droplet size and the existence of interfacial films that occupy water droplets. The presence of surfactants and native solids, such as silicates, calcium, and bicarbonate ions, and the residues of asphaltenes and resins, also recognized as emulsifiers, are added or occur naturally during crude oil production, enabling to promote the kinetic stability of emulsions.^7^ The stability of crude oil emulsions has gained interest among many oilfield researchers to invent different effective and relevant techniques to break it.^8,9^ According to researchers (Murtada Mohammed Abdulredha, Siti Aslina Hussain, and Luqman Chuah Abdullah, 2018),^1^ the basic reasons to form emulsions include the interaction between two immiscible fluids, the existence of emulsifying agents inside the crude oil, and diffusion of one liquid into another due to turbulent flow or mixing energy.

According to R. F. Lee,^10^ a stable emulsion consists of an aqueous phase, an oil phase, and an emulsifying agent. The emulsifying agent that exists at the oil interphase is commonly solid particles, surfactants, or mixtures of surfactants with other amphiphiles or amphiphiles polymers, which help facilitate the formation of the stabilized emulsion.^11^ The presence of surfactants in the oil–water interface facilitates the development of small droplets, which are significant in the preparation of emulsion. These surfactants enable to reduce the interfacial tension by assuming the surface-free energy changes during emulsification. The main functions of surfactants are promoting the formation of emulsion, generating a smaller droplet, and aiding the emulsion stability.^12^ The size of the organic or inorganic particles must be small enough, like a few microns or less, to ensure that the adsorption of the particles is interfacially active with the asphaltenes and resins from the crude. The additives that form around the dispersed droplets, such as surface-active agents, including particles and surfactants, can enhance the stability of an emulsion system.^13^

Besides, the formation of the stable emulsion is commonly stimulated by the amount of shear, which includes, flow-through reservoir rock, bottom-hole pump, turbulent flows, pressure change in choke valves and other valves during crude oil extraction, surface equipment, and gas bubbles released because of phase change.^1^ The effects of emulsions in the upstream crude oil production on the flow properties have been evaluated systematically. They disclosed many factors that influence the emulsion stability, such as mixing speed and duration, pH, temperature, and salt concentration. High mixing speed will produce a smaller size of emulsion droplets and increases interfacial area and droplet–droplet interaction.^14^ Several prime factors lead to the formation and stabilization of emulsions, such as temperature, droplet size, agitation, time, and types of emulsifying agents.^15^

Surfactants are categorized economically based on their usage, dissociation in water, and charge carrier.^16,17^ Hydrophilic–Lipophilic Balance (HLB) values measure the degree to which a surfactant is hydrophilic or lipophilic. HLB values of 0 indicate if the surfactant is a lipophilic molecule entirely, and 20 is ultimately a hydrophilic molecule. A surfactant is used to emulsify w/o emulsions usually in the range of HLB values of 3.5–6, meanwhile for o/w emulsions is in the range of 8–18. HLB values between 7 and 9 commonly represent the wetting agents.^18^ It is well known that nonionic surfactants increased by about 45% of the total production worldwide. The hydrophilic group shows a non-dissociating behaviour, thus, these surfactants do not ionize in an aqueous solution. As a result, they are suitable for other complex mixtures, as in many commercial products. The solvation of polyoxyethylene oxide groups provides solubility as in C_9_H_19_C_6_H_4_(OCH_2_CH_2_)_9_OH, nonylphenol ethoxylate. The characteristic properties of nonionic surfactants make them more effective. For example, it has ultra-low IFT and is a non-volatile, and environmentally safe surfactant. In addition, it is efficient in solubilization towards water-insoluble or moderately soluble organic compounds and can alter surface characteristics.^19^ The two main dissimilar parts are the hydrophilic head (water liking) and a hydrophobic tail (water disliking) in the amphiphilic molecule like surfactants. The hydrophilic part of the surfactant molecule can be positive (cationic), negative (anionic), neutral (nonionic), or zwitterionic (amphoteric). The effects of mixing two or more different emulsifiers may improve the formation, stability, and performance of oil-in-water emulsions.^20^ Recent work has focused on the effect of nonionic surfactants and/or particles on emulsion stability because of their associated ease of the process of adsorption on the particles through both hydrogen-bonding and hydrophobic interactions. The depletion of the surfactant from the aqueous phase is very noticeable in the emulsion stability.^21–24^

Yaghmur *et al.*^25^ reported a study on oil-in-water emulsion stability prepared with two different types of mixtures of nonionic emulsifiers, Span 80 and Tween 80. They highlighted the emulsifier concentration and hydrophile–lipophilic balance (HLB) value for the oil to provide an applicable emulsification technique for the formation of a stable oil-in-water emulsion. A synergistic effect was found to improve the stability of the emulsions prepared in the presence of both nonionic emulsifiers. N. H. Abdurahman *et al.*^26^ studied various factors influencing the stable crude o/w emulsions for Malaysian oil samples; Tapis crudes were stabilized by hydrophilic non-ionic surfactants. Several parameters were studied, such as the oil content, salinity of the water, speed of mixing the emulsion, duration of mixing, and pH of the aqueous phase. The result of this study revealed that the interfacial tension was reduced, and the stability of the emulsion increased when the surfactant concentration was increased. In addition, the stability of the emulsion also increased when the oil content, speed and mixing time, salt concentration, and pH of the aqueous phase of the emulsion increased. Furthermore, the effect of the pH of the water phase in the presence of non-ionic, cationic, and anionic surfactants was reported.^27^ They discovered that those non-ionic surfactants had shown a better improvement in the emulsion stability and oil/water phase compatibility compared to the other types of surfactants.

Due to the high stability of emulsion, the significant issue of the separation process is of practical interest. The crucial part of emulsion stability is to understand the mechanisms and factors that affect the stability to achieve the utmost separation goal and an excellent resolution of the various challenges associated with the production of crude oil emulsions.^3^ The stability of the produced emulsion is mainly caused by several factors, including water/oil ratio, emulsifiers present, the level of turbulence, temperature, pH, and brine composition. The characteristics of the produced emulsion may be different because of the changes in these factors.^28^

At present, the available research in understanding the stabilization emulsion mechanism is still in its infancy. Previous research studies have reported on the study of emulsions as the function of non-ionic surfactants as emulsifiers.^29,30^ RSM also has been used to investigate the performance of non-ionic emulsifiers during emulsion stability and the capability to break emulsions.^31–33^ Furthermore, several studies were reported on using Central Composite Design (CCD) for modelling the stability of the crude oil emulsion.^31,33–35^ There is an extensive approach to emulsions treatment to ensure the profitability of the industry. However, the use of non-ionic surfactants to study the stability of oil-in-water emulsions is less compared to other types of surfactants. The preferential properties of non-ionic surfactants are not having any negative or positive charge on the hydrophilic end and not ionising in an aqueous solution. Hence, these surfactants might prevent the erosion effect due to not responding to ions. Both non-ionic surfactants are also hydrophilic (water-soluble) and lipophilic (oil-soluble), making them favourable to act as emulsifiers. A water-soluble surfactant could form hydrogen bonds with the water droplets and facilitate the connections. There is no available prediction model in a previous study on the influence of synergized mixing of non-ionic surfactants in the stabilization of petroleum emulsions. The use of RSM to reveal the synergized mixing of non-ionic emulsifiers in modelling its long term-stability, especially in considering the mixing rate, mixing time and ageing time remains inadequately explained. More specifically, the type of surfactant, oil-in-water emulsions, and their interaction with colloidal particles are highlighted. A piece of fundamental knowledge about emulsifiers can present an innovative solution to distinguish the formation of a stable emulsion.

## 2 Materials and methods

### 2.1 Materials

PETRONAS Research Sdn supplied Malaysian offshore crude oil (Tapis). Bhd (PRSB), Bangi, in its pure form (99%). The crude oil was acquired from the test separator and used as the oleic phase. Before investigating the properties of the emulsion stabilized by the selected native solids, the utmost step of characterization of the crude oil was taken. The crude oil was treated by extracting its polar components (asphaltenes, resins, aromatics, and saturates) before preparing the emulsions. Deionized water with a resistance of ≥18.2 MΩ, pH 6.1 obtained from a PureLab Flex 3 purifier was used as the internal phase. Sodium chloride (NaCl) was purchased from R&M Chemicals. Nonionic surfactants sorbitan monooleate and polysorbate 80 (glycol), with HLB values 4.3 and 15, respectively, were used as emulsifiers.

The crude oil used in this study was Tapis crude oil. The crude oil field is located offshore Peninsular Malaysia, and it is a light crude oil, with API gravity ∼ 44° API, very sweet and easy to handle. Hence it is easier to treat the water—the lifting process of crude oil from a reservoir since it requires complex engineering procedures. Crude oil is seldom produced together with emulsified and free water. The emulsified water generates greater attention among researchers than the free water because the separation of the emulsified water from the crude oil needs adequate knowledge about the factors that cause its stability. In contrast, free water is quickly resolved by settling procedures. Before preparing the emulsion samples, the oil was characterized by following the crude procedures as listed in Table 1.

**Table 1:** Crude oil characterization procedures and methods

Parameters	ASTM standard or other methods	Equipment
SARA analysis	Group-type SARA analysis	High-performance liquid chromatography (HPLC)
Density and specific gravity measurement	ASTM D4052-96 (2002)	Anton Paar model DMA 4500 digital density meter
Viscosity	Electromagnetic concept	Electromagnetic viscometer (EV 1000)
Refractive index	ASTM D1747-09	DM40 Mettler Toledo refractometer

### 2.2 Methods

#### 2.2.1 Design of experiment-response surface methodology (RSM)

Both DoE and RSM are commonly used to statistically determine the significant factors (independent variables) in a model and provide a mathematical equation to develop a response surface model that can be employed to predict the outcome. The design parameters at which the responses achieve optimum conditions can be either maximum or minimum of the experimental design parameters. Response surface methodology (RSM) is a technique for achieving the optimum.^36,37^ It is an efficient design that gives the least number of experimental runs. RSM model consists of three main phases: (1) screening: the generation of data using the experimental design, which was aimed to find out the significant control that could give a huge effect on work practices, (2) modelling: experiments were designed to build a model of quality characteristic of interest (response) based on the control factors, and (3) optimization: the response was analyzed to discover the optimum conditions that could achieve the goal of the work.^38^ The aim of this part was to determine the optimal conditions to obtain maximum efficiency for water separation according to the developed models of RSM and optimize the response surface, which is influenced by several independent variables that lead to the emulsion formation.

**Fig. 1:**
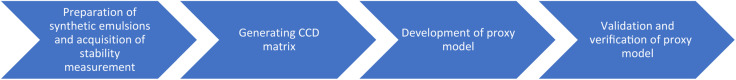
Steps for developing a proxy model for the prediction of emulsion stability.

In this study, the experiments were designed using commercial statistical software, Design Expert Version 10.0. The study design helped obtain optimal operating conditions to increase oil recovery with minor environmental damages and minimal production costs. This disclosed the significance of the individual parameters involved in producing stabilizing emulsions. The measure of stability was gauged from the amount of separated water from each emulsion measured at 60 °C and for seven days. The experiments were performed on the basis of the suggested number of experimental runs offered by the Central Composite Design (CCD). The design was applied to generate a mathematical model prior to obtaining the optimum combination of hexitol and glycol surfactants. The prediction of the optimum of the surfactants was according to the ability of the surfactants to release high water separation of the emulsions. The CCD design was also applied to evaluate the quadratic response surface. The step of the optimization procedure using CCD is shown in Fig. 1. The data obtained was utilized to develop a Response Surface Methodology (RSM) model.

Five independent variables were studied: concentrations of sorbitan monooleate, polysorbate 80, mixing rate, mixing time, and ageing time. The dependent variable (response) is the emulsion stability, ESI. Each independent variable (*x*_1_, *x*_2_, *x*_3_, *x*_4_, and *x*_5_) was varied numerically over three levels and coded as −1, 0, and 1. Analysis of variance (ANOVA) and regression analysis was conducted to determine how statistically consequential the model terms are and fit a regression correlation interacting the experimental data with the independent variables. Synthetic emulsions were prepared based on the response surface methodology using a central composite design (CCD). Forty-seven synthetic emulsions were prepared and observed following the parameters in Table 2, which shows the lower, middle, and upper experimental design parameters defined by −1, 0, and +1, respectively.

**Table 2:** Experimental design levels and parameters, symbols, and values

Variables	Coded levels			Unit
	−1	0	+1	
Concentration of hexitol	0.5	1	1.5	v/v%
Concentration of glycol	0.5	1	1.5	v/v%
Mixing rate	5000	10 000	15 000	Rpm
Mixing time	5	10	15	Min
Aging time	1	4	7	Days

Assuming the variations of *Y* (stability) obey an eight-parameter, second-order equation of the following type:

1 Stability = *β*_0_ + ∑*β*_*i*_*x*_*i*_ + ∑*β*_*ii*_*x*_*i*_^2^ + *β*_*ij*_*x*_*i*_*x*_*j*_

where *Y* is the response value predicted by the model; *β*_0_ is an offset value; *β*_*i*_, *β*_*ii*_, and *β*_*ij*_ are main (linear), quadratic, and interaction regression coefficients, respectively. The model analysis, lack-of-fit test, and coefficient of determination (*R*^2^) analysis serve the model's competence^39^ and suggested that the value of *R*^2^ should be at least 0.80 to demonstrate a good fitness of a response model. When the terms statistically show *p* > 0.05, they are non-significant variables. They would be excluded from the initial models, and the empirical data were refitted only to significant *p* < 0.05 variables to attain the final proxy model. The variable will be more significant if the absolute *t*-value becomes higher and the *p*-value becomes less.^34,40^

#### 2.2.2 Preparation of synthetic emulsions

Emulsions were prepared at 30 : 70 (w_oil_/w_water_) ratios, totaling 40 mL, to evaluate the effect of silica nanoparticles on the emulsion stability. Tapis crude oil was the dispersed phase, and deionized water was the continuous phase. The pH control of the aqueous phase was performed by adding HCl solutions to reach samples of pH 2 since the stabilizing agents generally have ionizing groups that differ based on the pH of the medium. The pH was chosen because O/W emulsions were stabilized using a mixture of particles and surfactants at pH 2. O/w emulsions were prepared and the emulsifiers used were nonionic surfactants, hexitol and glycol (0.5–1.5 v/v). The mixtures were homogenized with crude oil using IKA T18 Ultra Turrax T18 Homogenizer for 5 minutes. Then, the oil was mixed with deionized water at various mixing speeds and mixing times. The salinity was 10 000 ppm, and the system was operated at 60 °C.

In the oil refinery, the application of the thermal method for breaking emulsions is well utilized. According to Grace,^41^ oil's high viscosity can hold up more extensive water droplets than the lower viscosity of the oil. The viscosity of the stable oil emulsion is high, and the viscosity can be reduced at high temperatures and is usually significant in destabilizing and breaking the emulsions. Temperatures between 50 and 65 °C may effectively destabilize the emulsions, and the rate of oil droplet coalescence is directly proportional to the temperature.^14^ The prepared emulsions were filled in graduated plastic centrifuge bottles for stability analysis. Visual observation, also known as the bottle test, is an old and simplest method of observing gravitational separation in emulsions.^42,43^ Measurements of water resolution were performed at 30 minutes intervals for the first day and seven days.

#### 2.2.3 Emulsion stability measurement

The stability of the emulsion was determined using the static bottle test method by selecting the ratio of the volume of water-separated phase to the total volume of the mixture in a centrifuge bottle [emulsion volume ratio, *V*_e_ (%)] captured at different times at 60 °C.^44^ It is an old technique; however, it is advantageous and widely employed in the petroleum industry. Emulsion stability analysis was performed by placing the samples in the graduated tubes and kept in quiescent conditions. The separation of the water phase at the bottom of the container or emulsion samples with two phases with a surfactant layer in between was observed after a thorough breakdown.^45^ Then, the Emulsion Stability Index (ESI) was calculated as follows:2
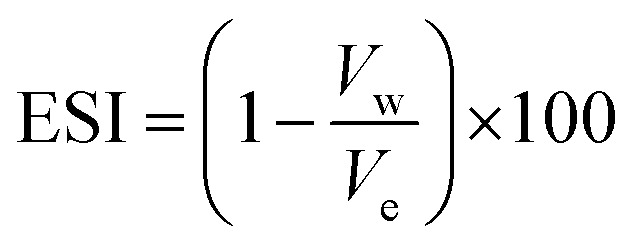
where *V*_e_ is the volume of the emulsions formed and *V*_w_ is the volume of the separated bottom layer (water) after the desired storage period.

## 3 Results and discussion

The characterization of crude oil was performed by determining the properties of SARA fractions, viscosity, density, and refractive index. In this study, the physical and chemical properties of crude oil were determined and are presented in Tables 3 and 4.

**Table 3:** Physical properties of crude oil

Characterization test	Results		
	15 °C	25 °C	60 °C
Viscosity, mPa s	2.0199	1.9686	1.3295
Refractive index	1.454	1.449	1.4303
Density, g cm^−3^	0.7983	0.7912	0.7656
API gravity, degree API	45.8		

**Table 4:** Chemical properties of crude oil

Characterization test	Results
Saturates (wt%)	76.00
Aromatics (wt%)	0
Resins (wt%)	5.00
Asphaltenes (wt%)	19.00

### 3.1 Effects of process variables – surfactant concentration sorbitan monooleate, polysorbate 80, mixing time, mixing rate, and ageing time

The formation of interfacial films and stability of emulsions stabilized by asphaltenes, resins, wax, and inorganic solids are widely studied.^46–48^

The preparation of emulsions, water, surfactants, and energy is required. When the emulsions with the emulsifier flow through the reservoir rock, bottom-hole perforations/pump, tubing, flow lines, production headers, valves, fittings, chokes, and surface equipment, they experienced different agitation speeds, which significantly lead to the formation and stabilization of emulsions. The mixing rate and time effects in addition to emulsifiers on emulsion stability are demanded and investigated in this study.

Emulsion stability was measured using the static bottle test method. This approach is certainly a standard method for investigating emulsion stability in the oil industry and is disciplined by gravity separation. The volume of the water resolved was observed over time and used as the determination of stability.^34,49,50^ The emulsions were prepared with 70 vol% water cut and stable for a seven-day experimental period. The stability of the emulsions in this study was observed as a function of the volume (%) of emulsified water emulsion separating at 60 °C and aged for a week (7 days). The percentage of separated water was determined as below: where:3
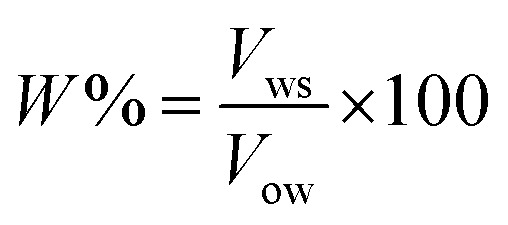
*W*% = percentage of water separated; *V*_ws_ = volume of water separated, in mL; and *V*_ow_ = volume of original water used to prepare the emulsion, mL.

In this experiment, two nonionic surfactants, hexitol (w/o emulsifier) and glycol (o/w emulsifier) were used as the emulsifiers. Nonionic surfactants are the most effective emulsifiers to emulsify emulsions and stabilize emulsions against flocculation and coalescence. In addition, emulsification and stabilization of the emulsions are more effective in the presence of nonionic surfactant mixtures.^51^

The effects of the selected process variables were determined using graphical and statistical data analysis using Design Expert Version 10, and the results are further discussed in this section. Response Surface Methodology (RSM) with the central composite design was used to determine the effects of independent variables on emulsion stability. The process parameters were: *X*_1_: concentration of hexitol (nonionic surfactant-oil soluble); *X*_2_: concentration of glycol (nonionic surfactant-water soluble); *X*_3_: mixing rate (in revolutions per minute); *X*_4_: time of mixing (in minutes) and *X*_5_: aging time (in days). An experimental range for each variable was chosen, and experiments at the midpoint of the design were conducted to make the valuation of pure error possible.

### 3.2 Regression analysis of the relationship between the independent parameters and emulsion stability

The ANOVA table categorizes the variability in emulsion stability (%) into several sections of the individual parameters. The ANOVA table shows the variability in emulsion stability into discrete divisions for the individual parameters. Furthermore, analysing the mean square upon an estimation of the experimental error demonstrates the statistical significance of the individual parameters. Five parameters have a *P*-value less than 0.05, showing that they are significantly different from zero at the 95.0% confidence level. The *r*-squared statistics justify that the model as fitted explains 88.14% of the irregularity of the emulsion stability. A value of 3.74 for the *F*-value indicates that the model is significant. The importance of “prob. > *F* being less than 0.00500 shows that the model term(s) are substantial. From the response stability, the ANOVA was tabulated as shown in Table 5. The essential terms are A (hexitol), B (glycol), C (mixing rate), E (ageing time), AC (product of hexitol and mixing rate), AE (product of hexitol and ageing time), and BC (product of glycol and mixing rate).

**Table 5:** Significant terms identified by ANOVA (quadratic model)

ANOVA for response surface quadratic model						
Analysis of variance table [partial sum of squares – type III]						
Source	Sum of squares	Df	Mean square	*F* value	*p*-Value (prob. > *F*)	
**Model**	**0.080**	**20**	**4.005 × 10** ^**−3**^	**5.27**	**<0.0001**	**Significant**
A-hexitol	4.108 × 10^−3^	1	4.108 × 10^−3^	5.40	0.0289	Significant
B-glycol	2.765 × 10^−4^	1	2.765 × 10^−4^	0.36	0.5522	
C-mixing rate	0.019	1	0.019	25.01	<0.0001	Significant
D-mixing time	2.485 × 10^−4^	1	2.485 × 10^−4^	0.33	0.5730	
E-aging time	1.900 × 10^−3^	1	1.900 × 10^−3^	2.50	0.1271	
AB	0.015	1	0.015	19.13	0.0002	Significant
AC	1.919 × 10^−3^	1	1.919 × 10^−3^	2.52	0.1253	
AD	7.914 × 10^−4^	1	7.914 × 10^−4^	1.04	0.3179	
AE	1.342 × 10^−6^	1	1.342 × 10^−6^	1.765 × 10^−3^	0.9668	
BC	4.197 × 10^−3^	1	4.197 × 10^−3^	5.52	0.0274	Significant
BD	7.816 × 10^−4^	1	7.816 × 10^−4^	1.03	0.3208	
BE	1.810 × 10^−4^	1	1.810 × 10^−4^	0.24	0.6301	
CD	2.524 × 10^−3^	1	2.524 × 10^−3^	3.32	0.0810	
CE	2.969 × 10^−3^	1	2.969 × 10^−3^	3.90	0.0598	
DE	8.561 × 10^−4^	1	8.561 × 10^−4^	1.13	0.2993	
A^2^	3.770 × 10^−3^	1	3.770 × 10^−3^	4.96	0.0356	Significant
B^2^	7.567 × 10^−4^	1	7.567 × 10^−4^	0.99	0.3285	
C^2^	4.342 × 10^−6^	1	4.342 × 10^−6^	5.708 × 10^−3^	0.9404	
D^2^	5.387 × 10^−5^	1	5.387 × 10^−5^	0.071	0.7924	
E^2^	4.454 × 10^−4^	1	4.454 × 10^−4^	0.59	0.4516	

The samples were prepared randomly to reduce inexplicable inconsistency in the outcomes due to systematic errors. All calculations and designs of the response were presented using electronic worksheets from Design Expert 10.0. The equation of the fitted model in terms of the coded factors is provided in the equation below:

4 Stability = 0.26835 − 0.22370*A*_1_ + 0.036996*A*_2_ + 1.66450 × 10^−5^*A*_3_ + 2.44305 × 10^−3^*A*_4_ + 3.55343 × 10^−3^*A*_5_ − 0.10120*A*_1_*A*_2_ − 3.56330 × 10^−6^*A*_1_*A*_3_ + 2.28856 × 10^−3^*A*_1_*A*_4_ − 1.62008 × 10^−4^*A*_1_*A*_5_ − 5.43533 × 10^−6^*A*_2_*A*_3_ − 2.22470 × 10^−3^*A*_2_*A*_4_ + 1.87462 × 10^−3^*A*_2_*A*_5_ − 4.08715 × 10^−7^*A*_3_*A*_4_ + 7.61943 × 10*−*^7^*A*_3_*A*_5_ − 3.88055 × 10^−4^*A*_4_*A*_5_ + 0.15626*A*_1_^2^ + 0.070005*A*_2_^2^ − 5.30283 × 10^−11^*A*_3_^2^ + 1.86789 × 10^−4^*A*_4_^2^ − 1.49203 × 10^−3^*A*_5_^2^

where: *A*_1_ = hexitol, *A*_2_ = glycol, *A*_3_ = mixing rate, *A*_4_ = mixing time, *A*_5_ = aging time (days).

The values of the variables are specified in their original units and are tabulated in Table 6. *E* symbolizes exponential, *i.e.*, 10^*n*^.

**Table 6:** Coefficient and units for the variables

Coefficient	Variables	Unit
*A*_1_	Hexitol	v/v%
*A*_2_	Glycol	v/v%
*A*_3_	Mixing rate	Rpm
*A*_4_	Mixing time	Min
*A*_5_	Aging time	Days

The equation in terms of the coded factors can be used to make predictions about the response for given levels of each element. In addition, the coded equation is helpful to determine the relative impact of the factors by comparing the factor coefficients.

**Fig. 2:**
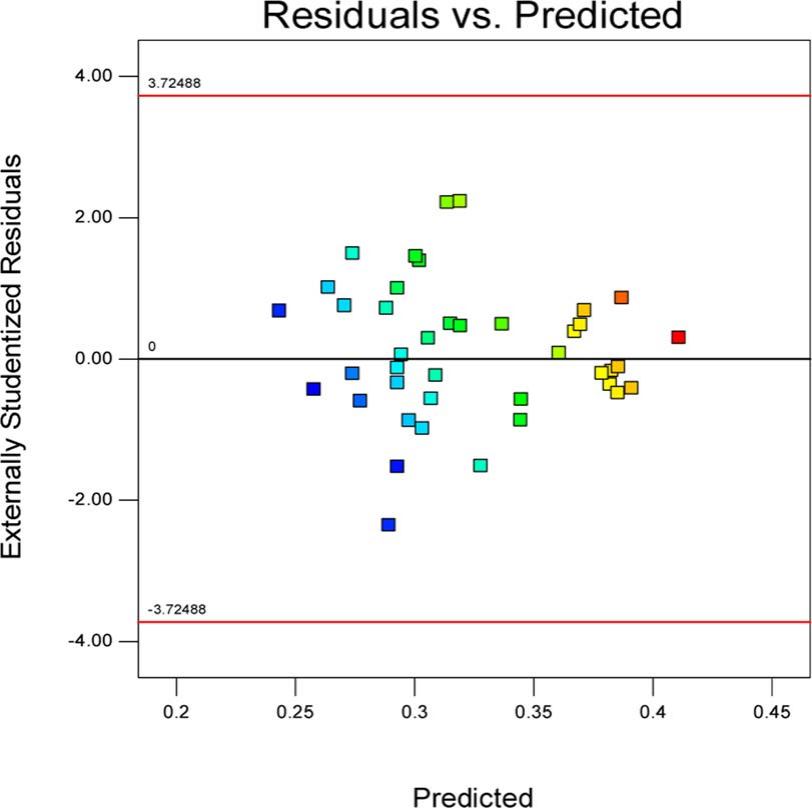
Externally studentized residuals.

Fig. 2 shows a plot of the residuals *versus* the ascending predicted response values. It is a visual inspection of the assumption of constant variance.

**Fig. 3:**
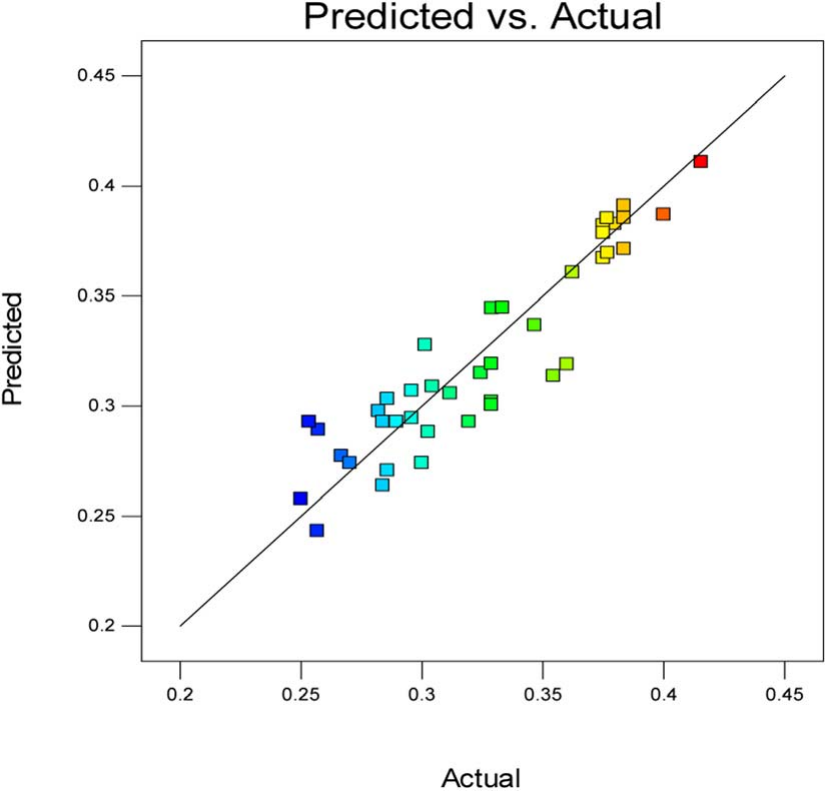
Predicted *versus* actual stability plot.

The diagnostic plot, which is a normal probability plot, was determined. The plot shown in Fig. 3 illustrates whether the residuals follow a normal distribution, in which the points will follow a straight line or not.

**Fig. 4:**
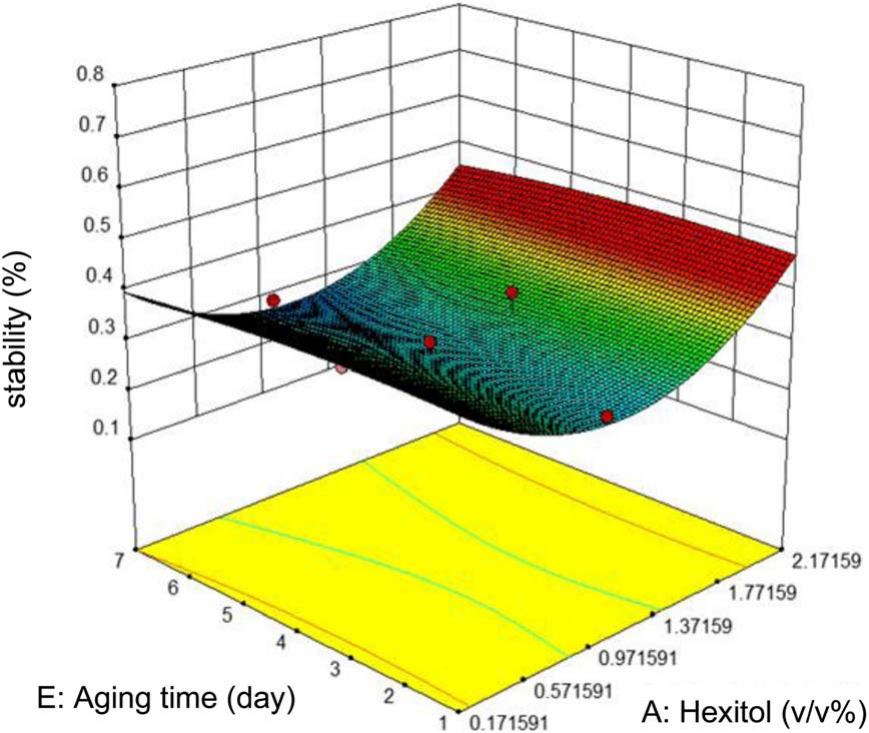
Combined effects of hexitol and aging time on emulsion stability.

**Fig. 5:**
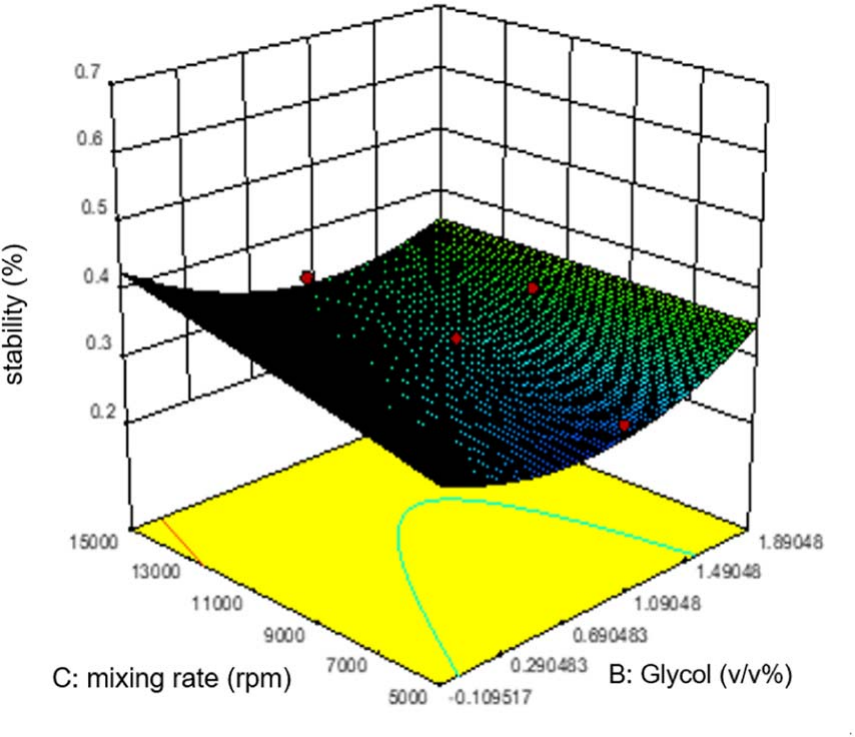
Combined effects of glycol and mixing rate on emulsion stability.

**Fig. 6:**
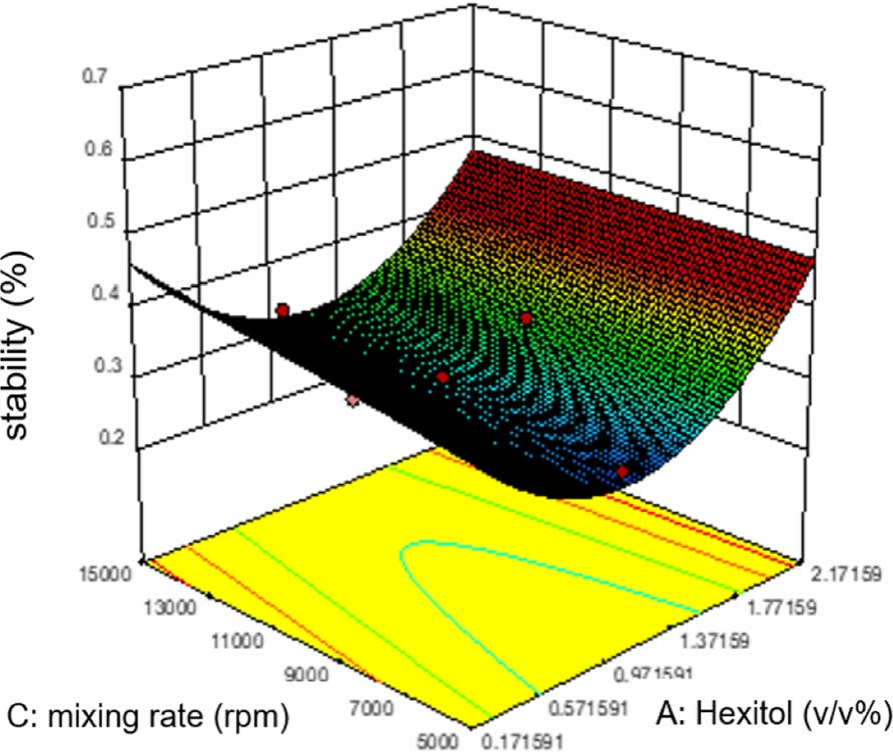
Combined effects of hexitol and mixing rate on emulsion stability.

**Fig. 7:**
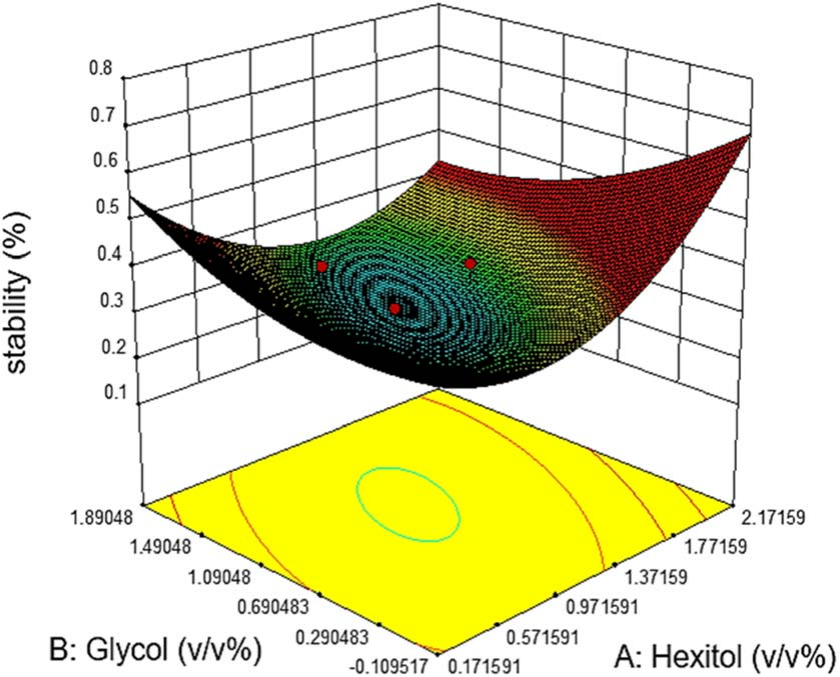
Response surface plots of the predicted stability as a function of hexitol and glycol.

Commonly, this plot is a random scatter with a steady high and low range of residuals across the predictions on the *X*_1_ axis. This plot is also significant for detecting outliers and runs with residuals outside the red lines on the plot. An outlier is not desired in a model because it is an observation that does not fit the model well. Based on the plot, no outlier can be seen as there is no point outside the red lines. The dependency of the independent parameters (factors) on the response (stability) can be demonstrated graphically. Fig. 4–7 show the mutual relationship plots.

The variation of stability with ageing time and hexitol at a concentration of 1.0 v/v% glycol, 10 000 rpm mixing rate, and 10 minutes mixing time is shown in figure. As shown, between the range of 0.97 and 1.37 v/v% hexitol, the emulsion stability is low, and the ageing time does not affect the stability of the emulsions. As hexitol increases, the emulsion stability increases. This observation indicates the decrease in the volume of the water released from the emulsions, interfacial activity, and interfacial coverage.

An energy input triggers the formation of emulsions at the surface, usually supported by shaking, stirring, or intensive dynamic and static mixing forces. This procedure is supported by the presence of stabilizing agents or surface-active substances (such as synthetic chemical surfactants and natural hydrocarbon surfactant: asphaltenes, resins, and solid mineral particles) that promote the stability of emulsions.^52^

As shown in figure, increasing glycol does not affect the emulsion stability as predicted by the ANOVA analysis. However, unlike the mixing rate, which is significantly based on the ANOVA analysis, the mixing rate between 5000 and 9000 rpm and 0.7–1.1 v/v% glycol shows lower stability of emulsions.

The relationship between the mixing rate and hexitol is plotted in Fig. 6. The effect of mixing time and the amount of hexitol is slightly different where at 1.8 v/v% concentration of hexitol, the emulsion shows the highest stability, but at 1.8 v/v% concentration of glycol, the emulsion does not demonstrate the most increased stability. The possible explanation for this observation is the competitive adsorption between the glycol and the hexitol molecules. Glycol is a water-soluble surfactant, which indicates that it would be helpful to form an oil-in-water emulsion than a water-in-oil emulsion. The adsorption of surfactants at liquid interfaces will reduce the interfacial tension and interfacial energy, increasing the emulsion stability, increasing surface elasticity, increasing electric double-layer repulsion (ionic surfactants), and probably growing surface viscosity.^53^ In the presence of glycol, there is an increase in the interfacial elasticity, which indicates that glycol prevents further interface, molecular organization, or multilayer development of the hexitol molecules at the oil–water interface.^30^

Emulsion stability increases when the concentration of hexitol and glycol increases, as shown in figure. The mixing of hexitol and glycol is one of the significant parameters in the emulsion stability supported by ANOVA. Mixed emulsifiers provide a higher film strength as determined by the static bottle test measurement, resulting in more stable o/w emulsions. However, the overall observation of the emulsion stability over seven days shows that the highest emulsion stability can be achieved at only 41.56%, and the lowest emulsion stability is 25.0%. The highest stable emulsion was obtained from 1.0 v/v% hexitol and 1.5 v/v% at 15 000 rpm mixing speed. It was observed that the high glycol concentration solubilizes hexitol, making it unobtainable to stabilize the emulsion.^29^

The effects of significant process variables and how they affect the emulsion stability are presented. From the static bottle test results, it can be concluded that the influence on the emulsion stability is mainly affected by the mixing rate, the concentration of mixing surfactants hexitol and glycol, and possible synergy between two different surfactants.

**Fig. 8:**
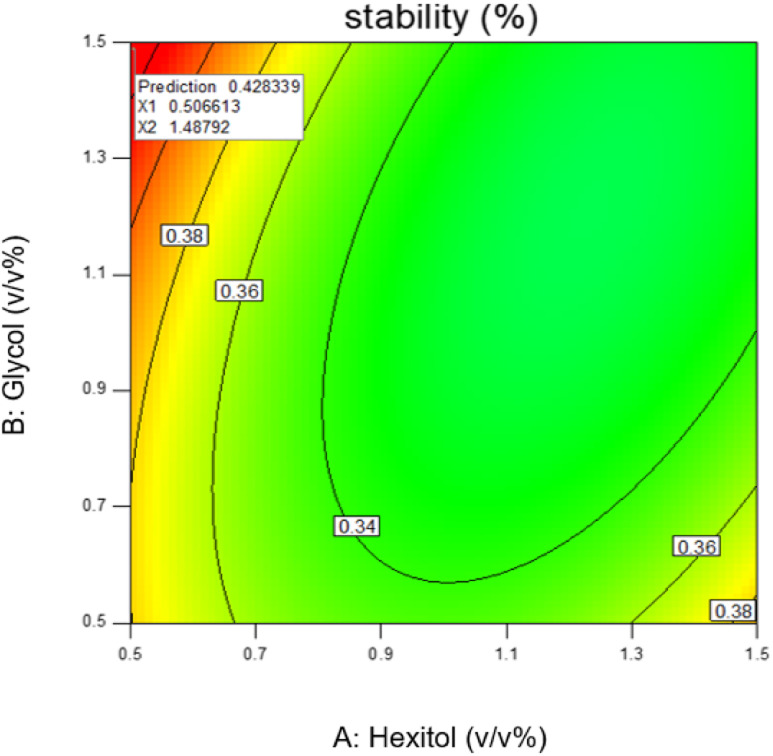
Numerical overlay plot of emulsion stability index in the presence of surfactants.

The most stable emulsion is with surfactants concentration (hexitol 0.5, glycol 1.5), mixing rate and duration of mixing 15 000 rpm and 5 min. As shown in Fig. 8, the red zone determines the highest emulsion stability, 42.83% of which was prepared from 0.5 v/v% of hexitol and 1.5 v/v% of glycol.

The stability (a measure of water released) in this emulsion is related to a high concentration of glycol (the water-soluble surfactants) and a low concentration of hexitol (the oil-soluble surfactant) and is connected to a synergy between both surfactants, which gave enhanced stability. The mechanism can be described by the formation of very low interfacial tensions through a synergistic effect that improves the strength of adsorption of the combination and the creation of supportive surfactant, which demonstrates higher strength (large effective energy barrier towards flocculation) to the o/w interface.^54^

### 3.3 Verification of RSM model

The optimum condition of the emulsification was used to check the suitability of equations for the prediction of the response. The optimized condition was obtained from the RSM model and validated by conducting experiments under optimum conditions. The optimum conditions were observed at 0.5 v/v% hexitol, 1.5 v/v% glycol, 15 000 rpm mixing rate in 5 minutes and seven-day ageing time (Table 7). The predicted response value at optimum conditions for emulsion stability is 41.56%. The experimental value of the emulsion stability was 40.36 ± 1.2%. The experimental data were in good agreement with the predicted value.

**Table 7:** Optimum conditions, experimental and predicted values of response at optimized conditions

Optimum conditions	Coded levels	Actual levels
Hexitol (v/v%)	−1	0.5
Glycol (v/v%)	+1	1.5
Mixing rate (rpm)	+1	15 000
Mixing time (min)	−1	5
Aging time (days)	+1	7
**Response**	**Predicted values**	**Experimental values**
Emulsion stability (%)	41.56	40.36 ± 1.2

## 4 Conclusions

This research used the bottle test method to investigate the effect of concentration of nonionic surfactants (sorbitan monooleate and polysorbate 80), the mixing rate, mixing time, and ageing time on the stability of emulsions. A proxy model based on the generated experimental design data was established. The CCD results were based on RSM-generated quadratic models for the experiments, and ANOVA analysis was performed to investigate the model's accuracy. The response surface approach provides a clear insight into the possible relationship between the parameters (*i.e.*, mixing rate, mixing time, ageing time, mixed surfactants) and the emulsion stability. Understanding the roles of the individual parameters in emulsion stabilization can present an innovative solution to the formulation of demulsification, specifically for the chemical demulsification method. ANOVA has shown that the synergy-mixing of both nonionic surfactants and the mixing rate is highly significant in emulsion stability since it strongly affects the interfacial film that encloses the dispersed phase. The synergy mixing of the surfactants adsorbed on the film provides a more rigid film between the fluid–fluid interfaces. The emulsion formulation containing 0.5 v/v% hexitol and 1.5 v/v% at 15 000 rpm mixing speed showed the best long-term stability after seven days. The mixed emulsifiers proved to have 41.56% emulsion stability, which also possesses almost 58.44% demulsification efficiency. However, the emulsion stability did not strongly affect the mixing time and ageing time of the emulsion under study.

## Author contributions

All authors contributed to the conception and design of the study. Material preparation, data collection, and analysis were performed by Sofiah Atirah binti Raya. The first draft of the manuscript was written by Sofiah. Ismail and Qayyimah commented on previous versions of the manuscript. Amirhilmi contributed on additional analysis required to address the comments and issues from the reviewers. All authors read and approved the final manuscript.

## Conflicts of interest

There are no conflicts to declare.

## Supplementary Material
